# A Three-Dimensional Electrospun Li_6.4_La_3_Zr_1.4_Ta_0.6_O_12_–Poly (Vinylidene Fluoride-Hexafluoropropylene) Gel Polymer Electrolyte for Rechargeable Solid-State Lithium Ion Batteries

**DOI:** 10.3389/fchem.2021.751476

**Published:** 2021-10-04

**Authors:** Donghuang Wang, Dan Cai, Yu Zhong, Zhao Jiang, Shengzhao Zhang, Xinhui Xia, Xiuli Wang, Jinagping Tu

**Affiliations:** ^1^ State Key Laboratory of Silicon Materials, Key Laboratory of Advanced Materials and Applications for Batteries of Zhejiang Province, and School of Materials Science and Engineering, Zhejiang University, Hangzhou, China; ^2^ Yangtze Delta Region Institute (Huzhou), University of Electronic Science and Technology of China, Huzhou, China

**Keywords:** poly (vinylidenefluoride-hexafluoropropylene), Li_6.4_La_3_Zr_1.4_Ta_0.6_O_12_ nanoparticles, three-dimensional, solid-state batteries, gel polymer electrolyte

## Abstract

Developing high-quality solid-state electrolytes is important for producing next-generation safe and stable solid-state lithium-ion batteries. Herein, a three-dimensional highly porous polymer electrolyte based on poly (vinylidenefluoride-hexafluoropropylene) (PVDF-HFP) with Li_6.4_La_3_Zr_1.4_Ta_0.6_O_12_ (LLZTO) nanoparticle fillers (PVDF-HFP-LLZTO) is prepared using the electrospinning technique. The PVDF-HFP-LLZTO gel polymer electrolyte possesses a high ionic conductivity of 9.44 × 10^–4^ S cm^−1^ and a Li-ion transference number of 0.66, which can be ascribed that the 3D hierarchical nanostructure with abundant porosity promotes the liquid electrolyte uptake and wetting, and LLZTO nanoparticles fillers decrease the crystallinity of PVDF-HFP. Thus, the solid-state lithium battery with LiFePO_4_ cathode, PVDF-HFP-LLZTO electrolyte, and Li metal anode exhibits enhanced electrochemical performance with improved cycling stability.

## Introduction

Nowadays, there has been a strong demand for developing rechargeable lithium batteries with high energy density and high safety for many applications, such as portable electronic devices, electric vehicles, and grid storage of electricity (Thangadurai et al., 2014; Manthiram et al., 2017; Zhao et al., 2020). The lithium metal batteries are extensively considered as the most promising candidates for next-generation rechargeable energy storage devices due to their high energy density (Fergus, 2010; Fan et al., 2018; Nair et al., 2019). However, the lithium metal batteries with flammable liquid organic electrolytes suffer severe safety issues of fire or explosion caused by lithium dendrite growth (Quartarone and Mustarelli, 2011; Fu et al., 2016; Mauger et al., d2017). Solid-state electrolytes (SSEs) can solve the safety issue raising from liquid electrolytes as they are nonflammable and have good mechanical strength to effectively suppress the lithium dendrite growth (Bachman et al., 2016; Li et al., 2020; Wang et al., 2020; Zhao et al., 2021).

In general, SSEs can be categorized into two major types: inorganic solid electrolytes (ISEs) and polymer solid electrolytes (PSEs) (Gao et al., 2018; Tan et al., 2020; Yuan et al., 2021). ISEs usually have excellent thermodynamic stability, wide electrochemical window, and high ionic conductivity (Li et al., 2018; Jiang et al., 2020). However, the high interfacial resistance between ISEs and electrodes caused by their rigid nature still impedes their application. PSEs alleviate drawbacks of ISEs as they possess high flexibility and good interfacial compatibility (Hazama et al., 2015; Cui et al., 2017; Jiang et al., 2018). The gel polymer electrolytes (GPEs), one type of the PSEs, have been a research hotpot, such as poly (ethylene oxide) (PEO) (Wu et al., 2018), poly (acrylonitrile) (PAN) (Zhou et al., 2015), poly (methyl methacrylate) (PMMA) (Liu et al., 2016), and poly (vinylidenefluoride-hexafluoropropylene) (PVDF-HFP) (Deng et al., 2018). Among the abovementioned polymer electrolytes, PVDF-HFP has attracted much attention due to its good thermal stability and mechanical strength (Fasciani et al., 2015). Nevertheless, PVDF-HFP usually suffers from low lithium-ion transference number and low ionic conductivity caused by the crystallization of polymer at room temperature.

To solve the stubborn problems, it is an effective and simple strategy to improve the absorbing ability of liquid electrolyte. A promising way is to directly introduce inorganic nanoparticles into the polymer matrix to decrease the crystallinity and facilitate the segment motion. Many inorganic fillers (such as SiO_2_, Al_2_O_3_, and TiO_2_) (Cao et al., 2013; Pandey et al., 2015; Blake et al., 2017) were doped into PVDF-HFP–based gel polymer electrolytes to improve the ionic conductivities with percolation effect. But these doping inert ceramic fillers may block the lithium-ion conducting routes because they cannot transfer the lithium ions. It is a feasible way to replace the inert ceramic fillers by some lithium ion conductors, such as Li_1.3_Al_0.3_Ti_1.7_(PO_4_)_3_ (LATP) (Xia et al., 2017), Li_0.33_La_0.557_TiO_3_ (LLTO) (Le et al., 2016), and Li_7_La_3_Zr_2_O_12_ (LLZO) (Liang et al., 2018). Among these lithium ion conductors, garnet LLZO has attracted much attention due to its outstanding stability with lithium metal anode and superior ionic conductivity (Zhang X. et al., 2017; Zhou et al., 2020). Another way is to prepare porous GPE membranes to improve the absorbing ability of liquid electrolyte (Wang et al., 2017; Zhang D. et al., 2017). However, the GPE membranes only own few holes synthesized by the traditional blade casting method, which leads to a bottleneck in forming fast ion channels (Farooqui et al., 2017; Shen et al., 2019). By contrast, electrospinning is a facile and effective method to prepare a porous nanofiber membrane, which constructs a three-dimensional (3D) network to uptake liquid electrolyte (Prasanth et al., 2014; Huang et al., 2015; Zhou et al., 2017). Li et al. prepared novel electrospun single-ion conducting polymer electrolytes, in which the single-ion conductive mechanism can facilitate the Li-ion transfer speed and obtain a high Li-ion transference number (Li et al., 2019).

Herein, we prepare a three-dimensional highly porous polymer electrolyte based on PVDF-HFP with Li_6.4_La_3_Zr_1.4_Ta_0.6_O_12_ (LLZTO) nanoparticle fillers by the electrospinning technique. This unique 3D hierarchical nanostructure possesses abundant porosity that promotes the liquid electrolyte uptake and wetting and favors the Li-ion transfer between the electrodes and electrolyte. In addition, the LLZTO nanoparticle fillers decrease the crystallinity of PVDF-HFP, thus improving the ionic conductivity and Li-ion transference number and ensuring the cycling stability and high rate performance.

## Result and Discussion

**FIGURE 1:**
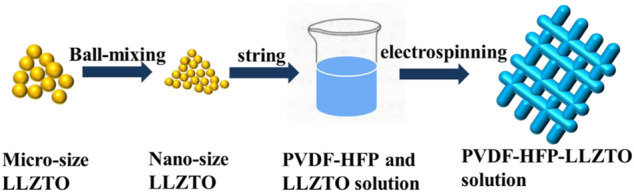
Schematic illustration of the synthesis of PVDF-HFP-LLZTO gel polymer electrolyte.

The PVDF-HFP-LLZTO gel polymer electrolyte was prepared by electrospinning, as shown in Figure 1. The microsize LLZTO powder was synthesized through a solid-state reaction and then was ball-milled into nanoparticles. And a proper amount of LLZTO was added into PVDF-HFP solution that was dissolved in *N*, *N*-dimethylformamide (DMF) with continuous stirring to form a uniform PVDF-HFP-LLZTO solution. The PVDF-HFP-LLZTO membrane was obtained by electrospinning and was activated by soaking the commercial liquid electrolyte.

**FIGURE 2:**
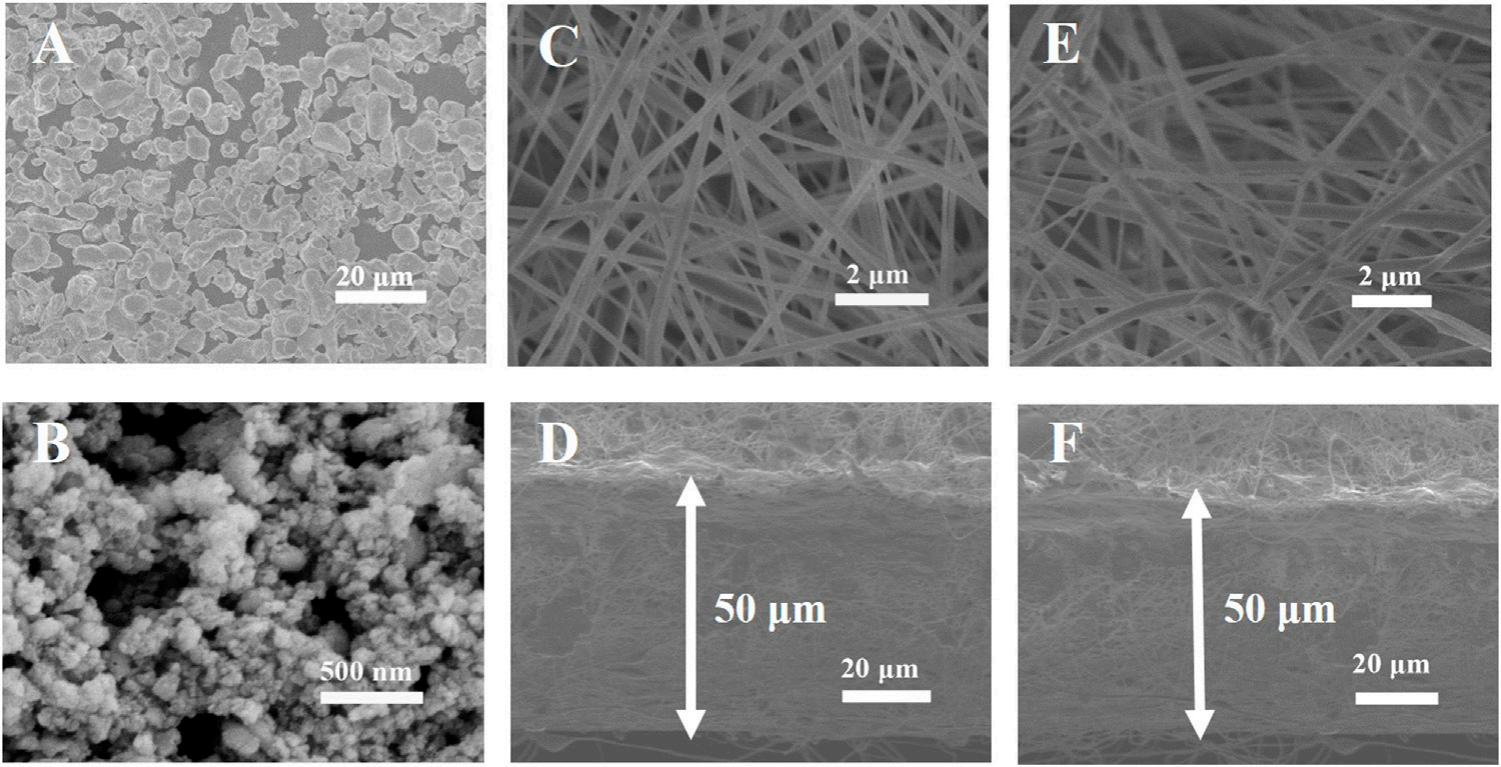
SEM images of **(A)** LLZTO before and **(B)** after ball-milling, **(C)** PVDF-HFP and **(E)** PVDF-HFP-LLZTO, cross-sectional SEM image of **(D)** PVDF-HFP **(F)** PVDF-HFP-LLZTO.

Comparison on the morphologies of the different samples is analyzed by SEM. As shown in Figures 2A,B, the LLZTO particles are in a diameter of about 5–10 μm before ball-milling. The LLZTO particles decrease to ∼50 nm after ball-milling. And the SEM images of PVDF-HFP and PVDF-HFP-LLZTO nanofiber membranes are shown in Figures 2C,E. It can be seen that tens of nanofibers with a diameter of ∼200 nm construct a three-dimension framework, which ensures a microporous architecture with sufficient void space to uptake liquid electrolyte. The PVDF-HFP-LLZTO membrane shows a similar morphology with the PVDF-HFP membrane, indicating that LLZTO nanoparticles have been doped into the nanofibers and the doping has no impact on the structure of the membranes. Figures 2D,F show a cross-sectional SEM image of PVDF-HFP and PVDF-HFP-LLZTO nanofiber membranes, and the thickness of the membranes is about 50 μm.

**FIGURE 3:**
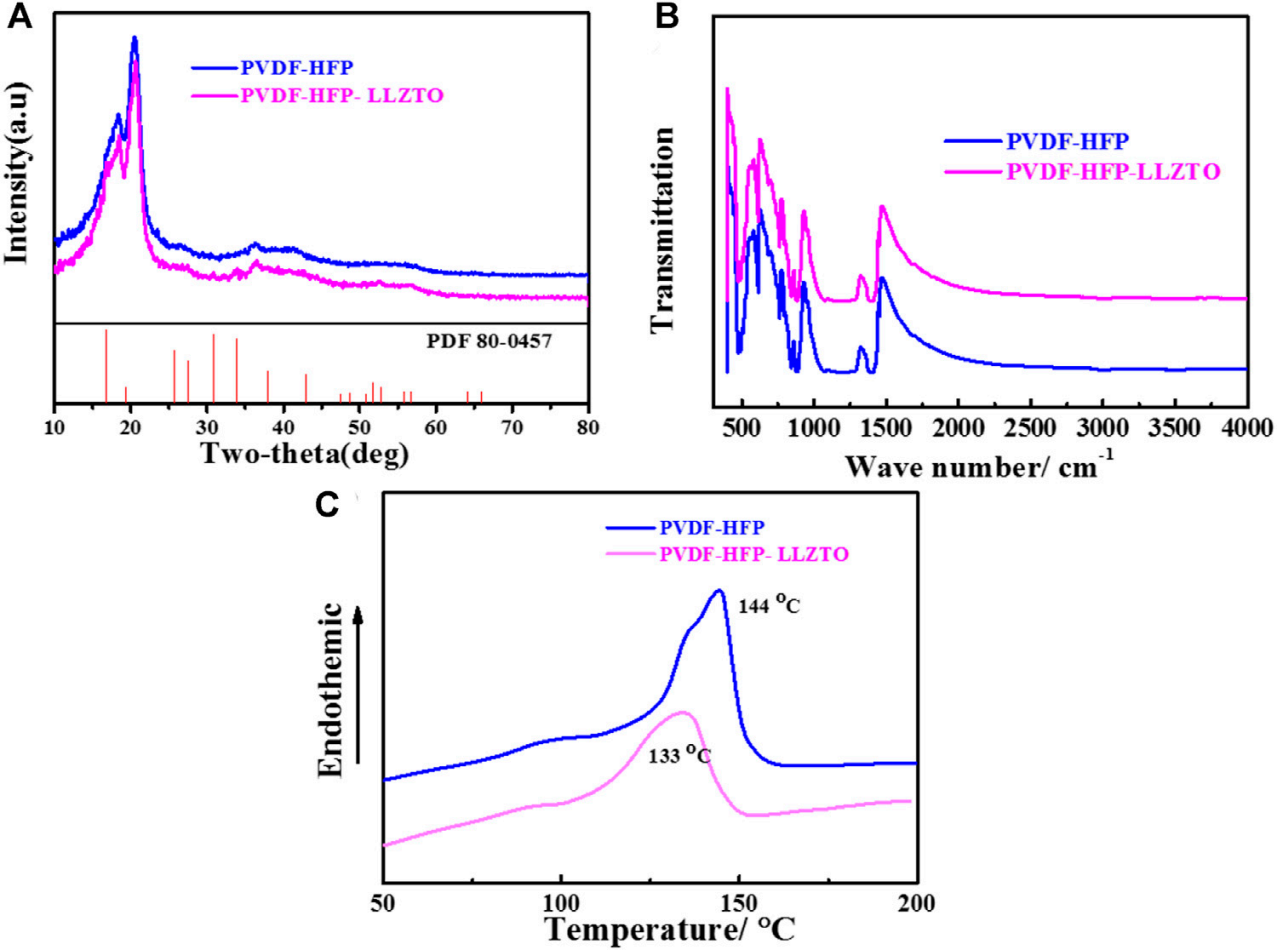
**(A)** XRD patterns, **(B)** FTIR spectra, **(C)** DSC patterns of PVDF-HFP and PVDF-HFP-LLZTO.

The phases and crystallinities of PVDF-HFP and PVDF-HFP-LLZTO membranes are investigated by XRD. As shown in Figure 3, three typical broad peaks of both samples were located at 18°, 20°, and 35°. In comparison, the PVDF-HFP-LLZTO membrane demonstrates weak diffraction peaks of LLZTO (JCPDS No. 80-0457), indicating that the LLZTO nanoparticles have been doped into the PVDF-HFP nanofibers (Ren et al., 2016). Additionally, the diffraction peaks of the PVDF-HFP-LLZTO membrane at 18°–20° are broadened than that of the PVDF-HFP membrane, which suggests that the crystallinity of PVDF-HFP decreases and the amorphous region expand, which can be ascribed to the introduction of LLZTO nanoparticles that disorder the regular long chain of PVDF-HFP (Le et al., 2016; Guo et al., 2017; Zhang X. et al., 2017). Figure 3B is the FTIR spectra of PVDF-HFP and PVDF-HFP-LLZTO membranes. After doping the LLZTO nanoparticles, the PVDF-HFP-LLZTO membrane shows similar peaks with the PVDF-HFP membrane, indicating that the combination of LLZTO nanoparticles and PVDF-HFP is a simple physical mixture without any chemical reaction, and the original foundation of PVDF-HFP matrix remained. DSC measurements are conducted to identify the thermal behavior of PVDF-HFP and PVDF-HFP-LLZTO membranes (Figure 3C). A sharp endothermic peak in both samples was observed, which indicated the melting temperatures (*T*_*m*_) of the polymer. The PVDF-HFP-LLZTO membrane displays *T*_*m*_ at 133°C, which is much lower than that of PVDF-HFP (144°C). It can be attributed to the LLZTO nanoparticle doping that decreases the degree of crystallinity of PVDF-HFP and enlarges the amorphous region in the PVDF-HFP matrix, as well as accelerate dynamic processes with plasticizing effect (Xia et al., 2017; Liang et al., 2018).

**FIGURE 4:**
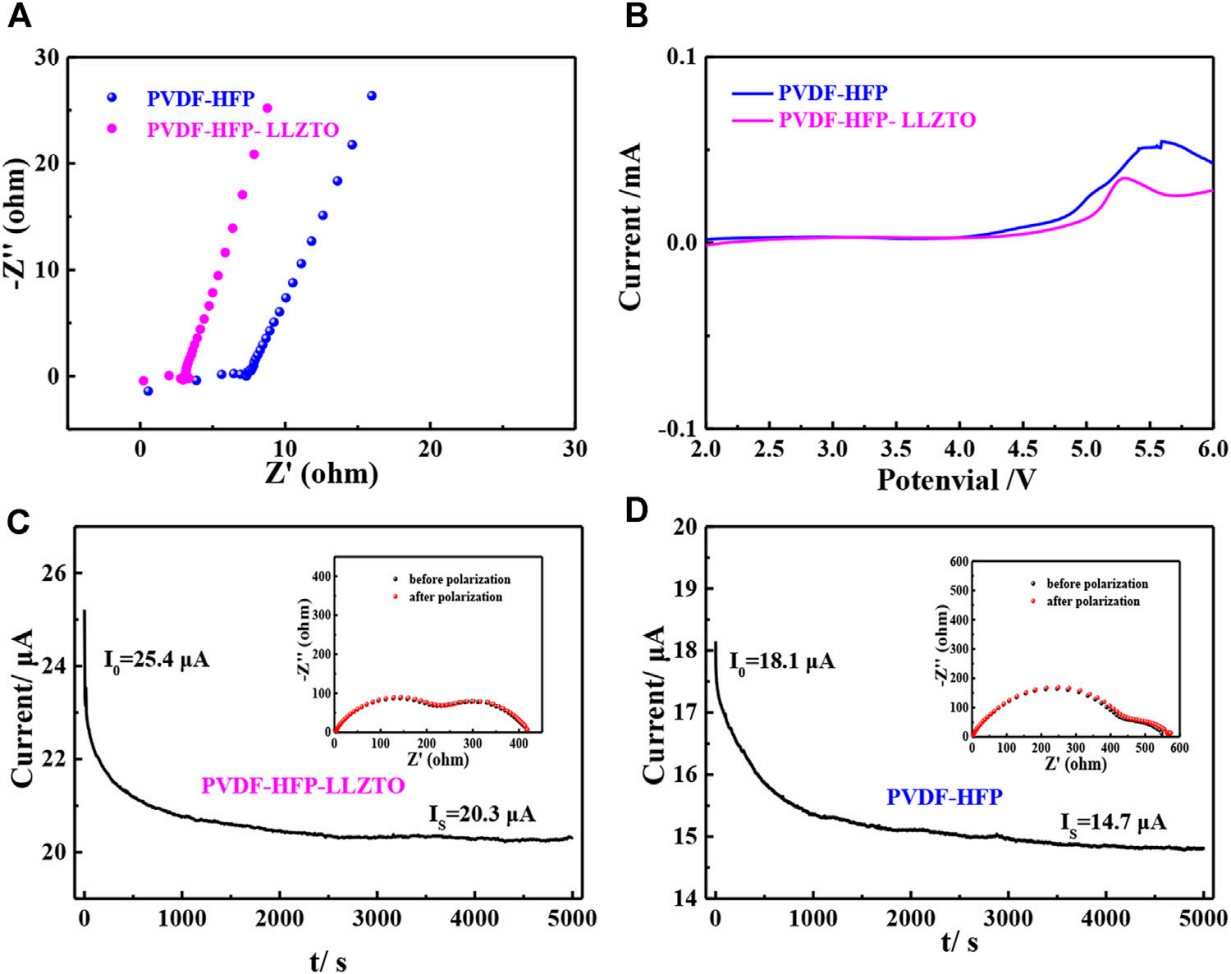
**(A)** EIS of SS/PH/SS and SS/PHL/SS cells, **(B)** LSV of Li/PH/SS and Li/PHL/SS cells, current–time curve of **(C)** Li/PH/Li and **(D)** Li/PH/Li cells at a DC polarization of 0.01 V, inset: the EIS of the cell before and after polarization.

Broadening the working voltage is an effective way to improve the energy density of batteries (Chen et al., 2016). To evaluate the electrochemical properties of PVDF-HFP and PVDF-HFP-LLZTO membranes, linear sweep voltammetry (LSV) is carried out by using Li//electrolyte//stainless steel cells. As shown in Figure 4B, the LSV curve of the PVDF-HFP-LLZTO membrane is smooth without a noticeable oxidation current before 4.6 V (vs Li/Li^+^), indicating an electrochemical stability window up to 4.6 V, which is higher than that of the PVDF-HFP membrane. The improved electrochemical stability of the PVDF-HFP-LLZTO membrane meets the requirement for the application of batteries with high voltage. Figure 4A shows the electrochemical impedance spectroscopy (EIS) of stainless steel//electrolyte//stainless steel, in which the semicircle at high frequency represents the impedance belonging to the electrolyte bulk. The ionic conductivity of the PVDF-HFP-LLZTO membrane at room temperature can be calculated as 9.44 × 10^–4^ S cm^−1^, which is higher than that of the PVDF-HFP membrane (4.04 × 10^–4^ S cm^−1^). However, the ionic conductivity of the PVDF-HFP membrane is still higher than that of the PVDF-HFP membrane synthesized by the casting method, as it builds a porous 3D network that has strong electrolyte absorption (Lim et al., 2015; Xia et al., 2017). The Li-ion transference number (*t*_*Li+*_) is an important parameter to reflect on the effective transportation of Li^+^ in solid electrolyte, which can be measured by a dc polarization combined with EIS of symmetrical Li//electrolyte//Li cells. As shown in Figure 4C, polarization increases the interfacial resistance from 220 to 225 Ω. Meanwhile, current varies from an initial value of 25.2 μA before polarization to a steady value of 20.3 μA. Therefore, the Li-ion transference number could be calculated based on the Bruce-Vincent equation, and the *t*_*Li+*_ value of the PVDF-HFP-LLZTO membrane is 0.66, while the t_*Li+*_ value of the PVDF-HFP membrane is 0.53. The remarkable improvement of the Li-ion transference number can be ascribed to the LLZTO nanoparticle doping, which enhances the mobility of Li^+^ and relax the local chain of polymer (Croce et al., 1998; Lin et al., 2005).

**FIGURE 5:**
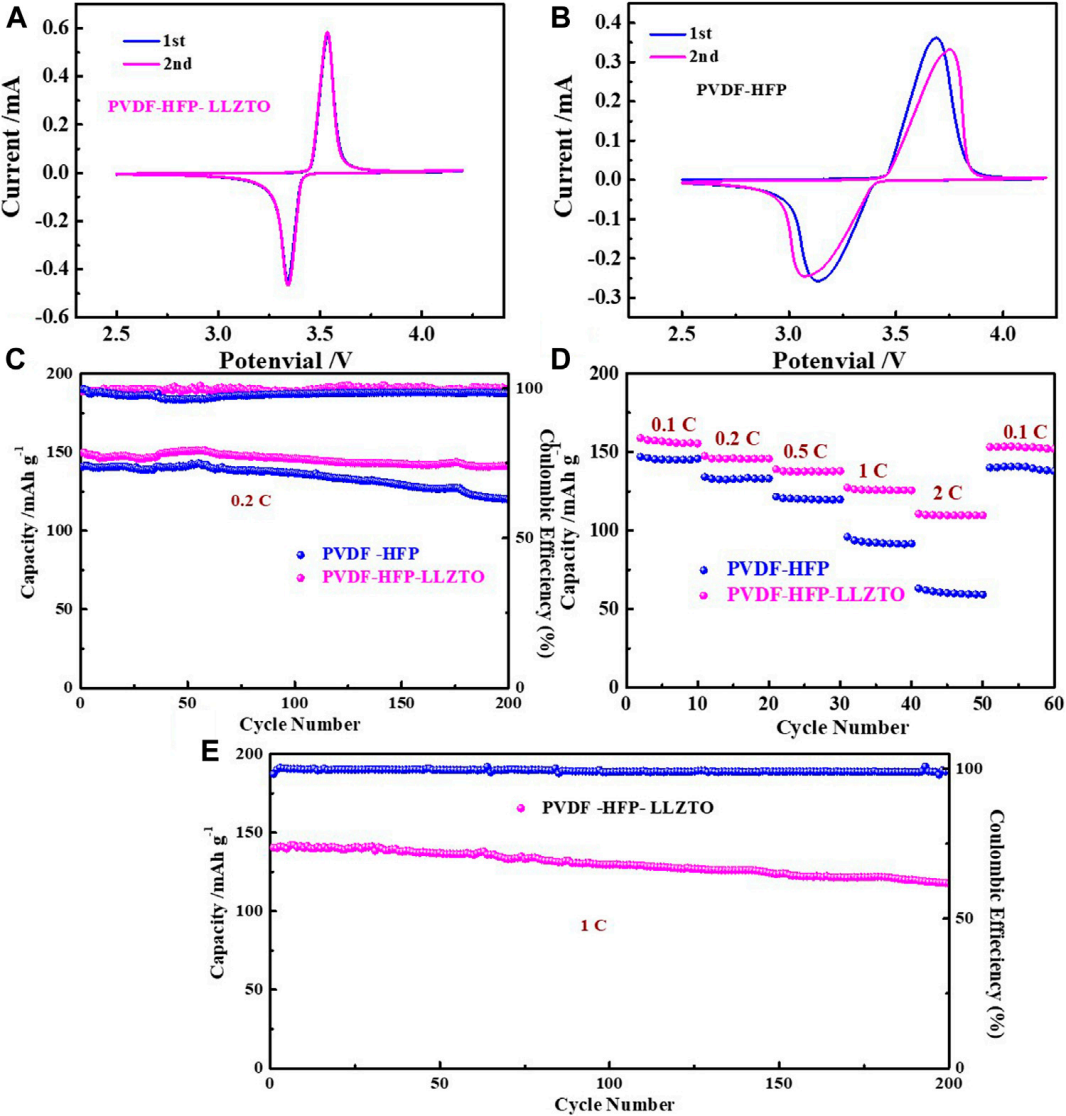
The CV curve of **(A)** LFP/PHL/Li cell, **(B)** LFP/PH/Li cell, **(C)** cycling performance at 0.2 C, **(D)** rate performance, and **(E)** cycling performance at 1 of LFP/PHL/Li and LFP/PH/Li cells.

To evaluate the electrochemical properties of PH and PHL membranes, we assemble LFP//PH//Li and LFP//PHL//Li cells. Figures 5A,B compare the CV curve of LFP//PH//Li and LFP//PHL//Li cells in the potential range of 2.5–4.2 V (vs Li/Li^+^) with a scanning rate of 0.1 mV s^−1^. There are two characteristic peaks of the LFP//PHL//Li cell appearing at around 3.53 and 3.34 V during anodic and cathodic scan. While the redox peaks of LFP//PH//Li cells are relatively broad, the anodic and cathodic peaks shift to 3.68 and 3.13 V, respectively. It demonstrates a decrease of cell polarization and an improved redox kinetics (Zhang et al., 2019).

To evaluate the rate capability of LFP//PH//Li and LFP//PHL//Li cells, the rate capability is tested at different current densities from 0.1 to 2 C. As shown in Figure 5D, the LFP//PHL//Li cell exhibits 158.9 mAh g^−1^, 147.4 mAh g^−1^, 139.1 mAh g^−1^, 127.4 mAh g^−1^, and 110.7 mAh g^−1^ at current densities of 0.1, 0.2, 0.5, 1, and 2 C, respectively. When the current density is converted from 2 to 0.1 C, the discharge capacity of the LFP//PHL//Li cell still can be recovered to 153.1 mAh g^−1^, suggesting a good reversibility of the LFP//PHL//Li cell. In contrast, the LFP//PH//Li cell only delivers 147 mAh g^−1^, 134.2 mAh g^−1^, 121.6 mAh g^−1^, 95.9 mAh g^−1^, and 63.3 mAh g^−1^ at current densities of 0.1, 0.2, 0.5, 1, and 2 C, respectively.

We also test long cycle electrochemical performance of LFP//PH//Li and LFP//PHL//Li cells. The cycling performance of LFP//PH//Li and LFP//PHL//Li cells is measured at a current density of 0.2 C (Figure 5C), and the LFP//PHL//Li cell exhibits a higher initial capacity of 149.6 mAh g^−1^ than the LFP//PH//Li cell (140.6 mAh g^−1^). It is worth noting that the capacity retention can be 94% even after 200 cycles and the Coulombic efficiency is almost 100% during the cycle. Additionally, high-rate long cycling life at 1 C of LFP//PHL//Li cells is shown in Figure 5E. The LFP//PHL//Li cell delivers the initial discharge capacities of 140.4 mAh g^−1^, and the capacity remains 117.6 mAh g^−1^, corresponding to a low capacity fading rate of only 0.089% per cycle. Therefore, the LFP//PHL//Li cell shows enhanced electrochemical performance which can be ascribed to the high ionic conductivity and transference number of the PVDF-HFP-LLZTO gel polymer electrolyte.

## Conclusion

In summary, we prepare a three-dimensional highly porous polymer electrolyte based on PVDF-HFP with Li_6.4_La_3_Zr_1.4_Ta_0.6_O_12_ (LLZTO) nanoparticle fillers using the electrospinning technique. This unique 3D hierarchical nanostructure with abundant porosity promotes the liquid electrolyte uptake and wetting, and favors the Li-ion transfer between the electrodes and electrolyte. And the LLZTO nanoparticle fillers decrease the crystallinity of PVDF-HFP; thus, the ionic conductivity and Li-ion transference number can reach 9.44 × 10^–4^ S cm^−1^ and 0.66. In addition, the LFP//PHL//Li cell exhibits enhanced electrochemical performance with improved cycling stability. This PVDF-HFP-LLZTO gel polymer electrolyte with high ionic conductivity and transference number demonstrates the potential application for solid-state batteries.

## Experimental Section

### Preparation of PVDF-HFP-LLZTO Gel Polymer Electrolyte

The PVDF-HFP-LLZTO gel polymer electrolyte (PHL) was prepared by electrospinning. First, the LLZTO powder was synthesized through a solid-state reaction according to the previous report. Then the microsize LLZTO powder was ball-milled into nanoparticles. 20 wt% PVDF-HFP was dissolved in *N*, *N*-dimethylformamide (DMF) with continuous stirring for 4 h. And a proper amount of LLZTO with 5 wt% was added into the solution with further stirring for 4 h to form a uniform solution. In the electrospinning process, the PVDF-HFP-LLZTO solution was electrospun onto the rotating aluminum collector with a flow rate of 1.5 ml h^−1^, while the voltage applied to the needle tip was set at 15kV with the distance of 20 cm from the needle to the collector. The obtained electrospun membrane was removed from the collector and evaporated at 60°C for 12 h. And the dry membrane was cut into 19 mm in diameter and was activated by soaking the commercial liquid electrolyte (1 M LiPF_6_ in EC/DEC 1: 1 by volume) for use. And the PVDF-HFP gel polymer electrolyte (PH) was prepared by the same method without LLZTO doping.

### Material Characterization

The morphology and microstructure of the samples were characterized by scanning electron microscopy (SEM, Hitachi S-4800). The crystal structure was investigated using X-ray diffraction (XRD, Rigaku D/max 2550 PC, Cu Kα). Fourier transform infrared spectra (FTIR) were characterized between 4,000 and 400 cm^−1^ with the Bruker VERTEX 70 FTIR spectrometer. Differential scanning calorimetry (DSC) was carried out on DSC Q100 from room temperature to 200°C under N_2_ flow at a heating rate of 5°C min^−1^.

### Electrochemical Measurements

To make the cathode, LiFePO_4_ (LFP), carbon black, and PVDF with a weight ratio of 8:1:1 were then dispersed in N-methyl-2-pyrrolidone (NMP). After magnetic stirring for 4 h, the homogeneous slurry was then cast on the Al foil, and the LFP cathode was dried at 80°C in vacuum for 12 h. The 2032 type cell was assembled with LFP as cathode, metallic lithium foil as anode, and the gel polymer electrolyte as electrolyte and separator. Cyclic voltammetry (CV) tests were carried out on CHI660E electrochemical workstation between 2.5 and 4.2 V at a scan rate of 0.1 mV s^−1^. The galvanostatic charge–discharge performance of the LiFePO_4_/electrolyte/Li cell at different rates were conducted between 2.5 and 4.2 V (vs Li+/Li) at room temperature. The galvanostatic test were carried out on a Land CT 2001A battery testing system at room temperature. The ionic conductivity of different membranes was carried out with the symmetric cell, and its value can be calculated by Eq. 2.

σ=L/(R×A), (1)

where *L* is the thickness of the electrolyte membrane, *A* is the electrode area, and *R* represents bulk resistance of the symmetrical stainless blocking cells, which could be measured on the Princeton multichannel electrochemical workstation from 10^6^ Hz to 10^–2^ Hz with an amplitude of 10 mV. The Li-ion transference number could be calculated by the Bruce–Vincent formula in Eq. 2.

$$t_{Li^{+}}=\frac{I_{s}\left(∆V-I_{0}R_{0}\right)}{I_{0}\left(∆V-I_{s}R_{s}\right)},$$ (2)

where *I*_*0*_ and *I*_*s*_ represent the initial and stable current which could be obtained from the DC polarization of symmetrical Li/electrolyte/Li cell, respectively. And *R*_*0*_ and *R*_*s*_ represent the impedance before and after polarization with a DC voltage (∆V = 10 mV), respectively.

## Data Availability

The original contributions presented in the study are included in the article/Supplementary Material; further inquiries can be directed to the corresponding author.
